# The complete chloroplast genome sequence of *Viburnum erosum* (Adoxaceae)

**DOI:** 10.1080/23802359.2019.1667919

**Published:** 2019-09-30

**Authors:** Jongsun Park, Yun Gyeong Choi, Narae Yun, Hong Xi, Juhyeon Min, Yongsung Kim, Sang-Hun Oh

**Affiliations:** a Infoboss Co., Ltd, Seoul, The Republic of Korea;; b InfoBoss Research Center, Seoul, The Republic of Korea;; c Department of Biology, Daejeon University, Daejeon, The Republic of Korea

**Keywords:** *Viburnum*, chloroplast genome, *Viburnum erosum*, Adoxaceae

## Abstract

*Viburnum erosum* is a deciduous shrub distributed in eastern Asia. As part of the systematic study to understand the phylogenetic relationship of *V*. *erosum*, we present the complete chloroplast genome of *V*. *erosum*. Its length is 158,624 bp and it has four subregions: 87,060 bp of large single-copy and 18,530 bp of small single-copy regions separated by a pair of inverted repeat regions of 26,517 bp each, including 129 genes (84 protein-coding genes, 8 rRNAs, and 37 tRNAs). Phylogenetic analyses show that *V. erosum* is sister to *Viburnum japonicum*, supporting morphological affinity of the two species.

*Viburnum erosum* Thunb. (Adoxaceae) is a deciduous shrub commonly distributed in southern China, Taiwan, Korea, and Japan and distinguished by having stipules, opposite leaves with extrafloral nectaries on the abaxial surface, and umbellate inflorescence (Choi and Oh 2019). It is closely related to *Viburnum dilatatum* Thunb., *Viburnum wrightii* Miq., and *Viburnum japonicum* (Thunb. in Murray) Sprengel. and intermediate forms are often found, resulting in taxonomic confusions (Hara 1983). As part of the systematic study to understand the phylogenetic relationship of *V*. *erosum*, we determined its complete chloroplast genome.

Sample of *V. erosum* was collected from Mt. Songgonsan (34°51'05.77''N, 125°18'18.01''E), Jeollanam-do, Korea (voucher in the herbarium of Daejeon University (TUT); OH 7522-04). Total genomic DNA was extracted from fresh leaves of *V. erosum* by using a DNeasy Plant Mini kit (QIAGEN, Hilden, Germany). Genome sequencing was performed using HiSeq4000 (Illumina, San Diego, CA) at Macrogen Inc., and *de novo* assembly was done by using Velvet 1.2.10 (Zerbino and Birney 2008), SOAP GapCloser 1.12 (Zhao et al. 2011), BWA 0.7.17 (Li 2013), and SAMtools 1.9 (Li et al. 2009). Geneious R11 v11.0.5 (Biomatters Ltd, Auckland, New Zealand) was used for genome annotation based on *V. japonicum* chloroplast genome (MH036493; Cho et al. 2018).

Chloroplast genome of *V*. *erosum* (GenBank accession is MN218778) is 158,624 bp (the GC-ratio is 38.1%) and has four subregions: 87,060 bp of large single-copy (36.4%) and 18,530 bp of small single-copy (31.9%) regions separated by a pair of 26,517 bp each of inverted repeats (43.0%). It contains 129 genes (84 protein-coding genes, eight rRNAs, and 37 tRNAs) with 18 genes (seven protein-coding genes, four rRNAs, and seven tRNAs) duplicated in the IR regions.

Complete chloroplast genomes from eight species of Adoxaceae and *Dipsacus japonicus* (NC_039668) as an outgroup, were included in the phylogenetic analysis using neighbor-joining and maximum-likelihood methods. Sequence alignment was conducted by MAFFT 7.388 (Katoh and Standley 2013) and phylogenetic trees were constructed by MEGA X (Kumar et al. 2018) with 10,000 and 1000 bootstrap repeats, respectively.

The phylogenetic tree shows that *V. erosum* forms a clade with *V. japonicum* (Figure 1), consistent with previous studies (Choi et al. 2018; Choi and Oh 2019). There are 18 single nucleotide polymorphisms (SNPs) and 36 insertions and deletions (INDELs) between *V. erosum* and *V. japonicum*, which is similar level between *Salix gracilistyla* and *Salix koriyanagi* (Xi et al. 2019). The levels of variation between the two *Viburnum* species is much lower than those of infraspecific variations found in other species, such as *Pseudostellaria palibiniana* (84 SNPs and 125 INDELs; Kim et al. 2019), *Pyrus ussuriensis* (121 SNPs and 781 INDELs; Cho et al. 2019), *Abeliophyllum distichum* (93 SNPs and 56 INDELs; Park et al. 2019), and *Goodyera schlechtendaliana* (1,794 SNPs and 827 INDELs; Oh et al. 2019). This study suggests that the rate of evolution of chloroplast genomes of *V*. *erosum* is slow. The complete chloroplast sequence of *V*. *erosum* determined in this study will be a useful resource for understanding the phylogeny of *Viburnum*.

**Figure 1.**
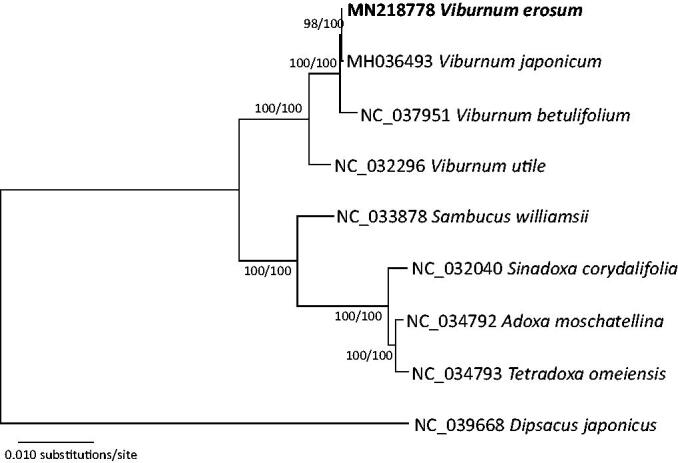
A neighbor-joining (with 10,000 bootstrap replicates) phylogenetic tree of eight complete chloroplast genomes of Adoxaceae, including *Viburnum erosum* (MN218778 in this study), *Viburnum japonicum* (MH036493), *Viburnum betulifolium* (NC_037951), *Viburnum utile* (NC_032296), *Adoxa moschatellina* (NC_034792), *Sambucus williamsii* (NC_033878), *Sinadoxa corydalifolia* (NC_032040), and *Tetradoxa omeiensis* (NC_034793) and an outgroup *Dipsacus japonicus* (NC_039668). The phylogenetic tree based on the maximum-likelihood method (with 1000 bootstrap replicates) was identical to the NJ tree. The numbers above the branches indicate the corresponding bootstrap support values from the neighbor-joining and maximum-likelihood methods.
